# Gender discrimination in surgical oncology: An in-house appraisal

**DOI:** 10.3389/fsurg.2022.939010

**Published:** 2022-07-12

**Authors:** Saneya Pandrowala, Shraddha Patkar, Deepa Nair, Amita Maheshwari, C. S. Pramesh, Ajay Puri

**Affiliations:** Department of Surgical Oncology, Tata Memorial Centre and Homi Bhabha National Institute, Mumbai, India

**Keywords:** gender, discrimination, surgical oncology, surgery, appraisal

## Abstract

**Introduction:**

Gender discrimination (GD) though rarely blatant, may present indirectly within a surgical department in the form of subtle inequities, differing standards, and bias. GD encompasses a wide spectrum including academic development, surgical opportunities and sexual harassment.

**Methods:**

We conducted an online survey to analyse the perceived incidence of GD in the surgical oncology department at a tertiary care cancer centre in India. The questionnaire consisted of 15 questions and was mailed to the entire department including trainees and faculty. Anonymity was maintained while collecting the data only of the participants' gender and whether they were faculty or trainee. Collated responses were analysed using proportions.

**Results:**

The questionnaire was sent out to 200 recipients of whom 56% (112/200) responded *via* an online survey. Respondents included 84% of faculty (42/50) and 46.6% of trainees (70/150). GD was perceived by 28% of female trainees (7/25) as compared to 6.6% of male trainees (3/45), whereas amongst faculty, GD was perceived by 26.6% of female faculty (4/15) compared to 14.8% of male faculty (3/27). Approximately 13% of our trainees and 12% of our faculty mentioned that GD affected their professional performance or mental well-being. GD was experienced in terms of work experience and opportunities by a majority of trainees (13%) and faculty (9.5%). There was a significant lack of awareness about recourse to an institutional grievance committee by trainees (47%) compared to faculty (14%). About 7% of trainees and 12% of faculty acknowledged that they may have been responsible for intentional/unintentional GD.

**Conclusion:**

Gender discrimination can present in subtle or overt fashion in surgical departments and requires active sustained efforts to allow both genders to feel equally empowered. Establishing a system to objectively evaluate gender equity while avoiding stereotyping for certain roles can help minimize GD.

## Introduction

Gender stereotyping is ingrained in society to such an extent that women get subconsciously habituated to gender discrimination (GD). This is especially true in surgical fields, where a masculine, confident and competitive stereotype is considered the norm and celebrated (1, 2). Breaking the glass ceiling requires determination, courage and patience; it took 26 years from the first physician to the first woman surgeon in India (3). Planning a surgical career for women physicians is challenging, requiring planned sacrifices both professionally and in their personal lives. There is additional pressure to excel in a field in which they are traditionally under-represented (4). In spite of working as much as their male counterparts while balancing professional and family life (5, 6), women face GD at various levels of their surgical careers from residency to academic positions to salaries (7). GD can present as unequal surgical opportunities, lack of respect from co-workers, differences in pay equity, imbalanced leadership roles and fewer academic opportunities (8–10). Although not a direct form of GD, women surgeons get fewer surgical case referrals as compared to their male counterparts (11).

GD is not necessarily restricted to women and can be perpetrated and experienced by both men and women (10). Besides impacting the individual directly affected, GD may also affect others who witness such misconduct. While having long term effects on the individuals concerned, it also creates an undesirable work environment for faculty, trainees and all involved. While not implying that female surgical trainees need to be treated differently or “delicately” (12), the importance of a gender diverse workplace with equal roles and opportunities is increasingly being recognized. Identification of GD is the first step towards changing preconceived notions, thought processes and attitudes. Literature on GD in India is limited and so is our understanding of the situation in the country (13, 14). We initiated an online survey among our surgical trainees and faculty to assess whether GD was prevalent in the surgical oncology department.

## Materials and methods

We conducted a cross-sectional study at the Tata Memorial Hospital, a tertiary level comprehensive cancer centre in Mumbai, to identify if GD was prevalent in the department of surgical oncology. Faculty and trainees were invited to participate in an online survey. Participation was voluntary and anonymized. The survey was created after an online review to identify questions relevant to GD which led to pooling of 30 most relevant questions. A panel comprising of faculty and trainees then distilled these to 11 questions with multiple choice answers, and its perceived impact on the participants’ surgical career (Table 1). The online survey was sent *via* email in the form of a Google Survey sheet to surgical trainees and faculty. Two reminders *via* individual email were given 10 days apart and once on a common trainees’ group. The survey was closed for responses after 2 weeks. To gather insights on perceptions and suggestions for improvement, we provided an option of free text entry for all participants. Besides the responses; gender, age and position at which the respondent was currently employed were also recorded. The department comprises of 50 faculty members (33 males – 66% and 17 females- 34%) and 150 trainees (113 males- 75% and 37 females- 25%).

**Table 1:** Responses of trainees and faculty to the questionnaire.

Sr. no	Questionnaire	Trainees		Faculty	
		Female trainees (*n* = 25)	Male trainees (*n* = 45)	Female faculty (*n* = 15)	Male faculty (*n* = 27)
1.	Age group				
	<30 years	6 (24%)	10 (22.3%)	0	1 (3.7%)
	30–50 years	19 (76%)	35 (77.7%)	13 (86.6%)	24 (88.8%)
	>50 years	0	0	2 (13.4%)	2 (7.5%)
2.	Do you receive equal developmental opportunities or responsibilities as compared to your opposite gender?				
	No	6 (24%)	3 (6.6%)	1 (6.6%)	2 (7.5%)
	Yes	19 (76%)	42 (93.4%)	14 (93.4%)	25 (92.5%)
3.	Do you receive equal surgical exposure as compared to your opposite gender?				
	No	6 (24%)	3 (6.6%)	2 (13.4%)	1(3.7%)
	Yes	19 (76%)	42 (93.4%)	13 (86.6%)	26 (96.3%)
4.	Have you faced gender discrimination?				
	No	18 (72%)	42 (93.4%)	11 (73.4%)	23 (85.2%)
	Yes	7 (28%)	3 (6.6%)	4 (26.6%)	4 (14.8%)
5.	If yes, in the form of				
	Work experience and opportunities	9 (13%)		4 (9.5%)	
	Given clerical jobs only	–		1 (2.4%)	
	Objectionable behavior	1 (1.4%)		1 (2.4%)	
	NA	–		2 (4.7%)	
6.	If yes, it was from				
	Faculty	4 (16%)	3 (6.6%)	2 (13.3%)	4 (14.8%)
	Trainees	1 (4%)	–	2 (13.3%)	–
	Patients	2 (8%)	–		
7.	Did it affect your professional performance?				
	No	5 (20%)	–	2 (13.3%)	3 (11.1%)
	Yes	2 (8%)	3 (6.6%)	2 (13.3%)	1 ((3.7%)
8.	Did it affect your mental well-being?				
	No	1 (4%)	2 (4.4%)	1 (6.6%)	2 (7.4%)
	Yes	6 (24%)	1 (2.2%)	3 (20%)	2 (7.4%)
9.	Are you aware of an institutional grievance/ complaints committee?				
	No	13 (52%)	20 (44.4%)	1 (6.6%)	5 (18.5%)
	Yes	12 (48%)	25 (55.6%)	14 (93.4%)	22 (81.5%)
10.	Have you approached the committee or brought to the notice of the department/hospital authority any issues related to gender discrimination?				
	No	7 (28%)	3 (6.6%)	1 (6.6%)	4 (14.8%)
	Yes	–	–	3 (20%)	–
11.	With the benefit of hindsight, have you been responsible (unintentional/intentional) at any time for gender discrimination?				
	No	15 (60%)	37 (82%)	7 (47%)	25 (92%)
	Yes	0	1 (2%)	1 (7%)	0
	Maybe	3 (12%)	1 (2%)	4 (27%)	0
	Cannot Say	7 (28%)	6 (14%)	3 (19%)	2 (8%)

### Statistics

Responses were entered into a Microsoft Excel v.2016 datasheet, maintaining anonymity and only revealing gender and the position at which the respondent was currently employed. Results were analyzed based on the gender of the respondents with descriptive statistics using percentages as overall. Categorical variables were summarized as numbers with proportions.

## Results

The online questionnaire was sent out to 200 recipients out of whom 112 (56%) responded. Amongst faculty 42/50 (84%) responses were recorded with 27/33 (82%) from male faculty and 15/17 (88%) from female faculty. Amongst trainees 70/150 (46.6%) responses were recorded with 45/113 (48.6%) from male and 25/37 (67.5%) from female trainees. Most trainees (>75%) and faculty (>85%) were in the 30 to 50-year age group in both genders.

### Responses of trainees

The responses of 25 (67.5%) female and 45 (48.6%) male trainees to the survey are shown in Table 1. With regards to opportunities to develop and surgical exposure, 6 (24%) women and 3 (6.6%) men felt that they received unequal opportunities as compared to the opposite gender. Nearly one-third female trainees (28%, 7/25) perceived that they had been discriminated on the basis of their gender while only 6.6% (3/45) male trainees felt similarly. Amongst the 10 (14%) respondents perceiving GD, five experienced disturbed professional performance, with mental well-being being affected in three trainees. Four trainees, who had their mental well-being affected due to GD, did not feel it reflected on their professional performance. Unequal work experience and opportunities was the most common reason to perceive GD (13%, 9/70) amongst trainees. Seven trainees perceived that faculty was responsible for GD. There was a lack of awareness about recourse to an institutional grievance committee in 47% of trainees. Five trainees (7%) considered that they themselves might have been responsible for GD intentionally or unintentionally; two of these mentioned experiencing GD during this survey.

### Responses of faculty

Differing developmental opportunities based on gender was perceived by one (6.6%) female faculty as compared to 2 (7.5%) male faculty whereas absence of equal surgical exposure was perceived by 2 (13.4%) female faculty as compared to one (3.7%) male faculty. Overall, GD was perceived by 4 (26.6%) women and 4 (14.8%) men which was mostly in the form of work experience and opportunities (*n* = 4) and was from a co-faculty (*n* = 6). GD affected professional performance of 3 (7.1%) consultants and had an impact on their mental well-being. Two faculty members felt GD had a bearing on their mental well-being without affecting professional performance. There was a lack of awareness about recourse to an institutional grievance committee in 14% of faculty. Five faculty members (12%) thought they might have been responsible for intentional/unintentional GD and these included two of the faculty who mentioned experiencing GD during this survey.

## Discussion

The results of our survey show that GD is perceived by both male and female members in our department, but was clearly higher among female trainees and faculty (28% and 26.6% female trainees and faculty, compared to 6.6% and 14.8% male trainees and faculty respectively). Trainees across both genders largely equated GD with developmental opportunities or surgical exposure. However, amongst faculty, GD was perceived for reasons besides developmental opportunities or surgical exposure.

Being the largest tertiary cancer center in India, our surgical oncology department is one of the largest in the country with 50 faculty and 150 trainees in 2021. The proportion of female faculty has increased manifold from 7/35 (20%) in 2012 to 17/50 (34%) in 2021, highlighting the fact that an increasing number of women are embarking on a career in surgical oncology and capability and experience, rather than gender are the criterion for selection of faculty. The institute prides itself on nurturing a gender-neutral, bullying-free workspace for our trainees and faculty and we strive to give equal opportunities to all, irrespective of gender, with merit being the yardstick to evaluate and determine capability and efficiency. Recognizing the need to continue to maintain a gender-neutral workspace and help create awareness of this issue, we conducted this anonymous survey to assess the ground situation in our department.

In the United States, women constitute >50% of the current medical school graduates but this is not reflected in surgical residency (15, 16). There are a number of barriers for women seriously considering a surgical career as their first option. Due to the masculine surgeon stereotype and constant stress to overachieve, women surgeons perceive discrimination as high as 89% even in high income countries (10, 17). Based on a survey from the United States, 87% women perceived GD in medical school, 88% in residency, and 91% in practice (10). In low- and middle-income countries more than half of female medical students do not proceed to specialty training (18, 19). Our survey results showed much lower proportions of surgeons who perceived GD. The reasons for this could be many. Surgical oncology in India is typically pursued after post-graduation in the broad surgical specialties. Trainees in our department of surgical oncology have completed three years of a basic surgical training prior to enrolling in surgical oncology, and hence our cohort is different from undergraduate medical students or general surgery trainees in their initial years. Apart from a different cohort of trainees, our department tries to build a gender-neutral workspace with minimal hierarchy which is emphasized from the very first day of joining to all trainees. We have two resident representatives from male and female genders to allow effective communication from trainees to the head of department. They are encouraged to discuss any decision they have a difference of opinion on and provide possible solutions to problems faced.

The factors responsible for gender bias include workplace challenges, assessment of credibility and objectification by patients, colleagues and self (20). GD may also manifest as workplace harassment of female surgeons by staff, patients and colleagues. This can range from inappropriate verbal remarks to physical contact (20, 25–27). The most important issues faced by women surgeons include ineffective mentorship, gender stereotypes, work-family issues and a perceived lack of belonging (18, 21–24). Perceptions also differ based on country of origin. Most reported studies on GD have emanated from high-income countries especially in the last five years. GD combined with lower levels of respect and constant objectification can result in psychological effects of GD leading to low self-esteem and confidence affecting the quality of work performed, which may ultimately culminate in burnout and attrition (20, 26–28). Approximately 13% of our trainees and 12% of our faculty mentioned that GD affected their professional performance or mental well-being. This survey also helped trainees and faculty introspect, as 7% of our trainees and 12% of our faculty thought they might have been responsible for intentional/unintentional GD.

Women face discrimination in the workplace in every field right from hiring to promotions to differences in pay and career opportunities (29). Amongst the Fortune's top 500 companies only 37 of the CEOs were women in 2020 which was an all-time high (30). Women held 38% of managerial positions as compared to 62% for men in 2020 (30). The first female CEO of General Motors, USA was paid less than half compared to her male predecessor (29). Beyond blocked opportunities and reduced wages, the position of a level of authority is also accompanied by an unsupportive environment which makes it difficult to work effectively (31). GD has been recently condemned publicly which is seen in all fields of medicine to be experienced more by women (32, 33), however, surgical fields pose a different challenge due to the male stereotype deeply rooted in the minds of patients, nursing staff and colleagues.

Identifying the presence of GD without recommending solutions is a job “half done”. Possible avenues include.

•  Basic minimum surgical requirements ensuring equal surgical opportunities

Trainees in surgical specialties allow themselves to be proved “worthy of their operative training”. Hence, introducing an objectivity with every rotation requiring a basic minimum surgical requirement to be completed at the end of training period will reduce bias and enable providing equal surgical opportunities. Though this system requiring a minimum number of performed and assisted surgeries per rotation in a surgical sub specialty does exist in our department, it is important to regularly audit and ensure that this system is functional.

•  Awareness of institutional grievance/ complaints committee

While most of the faculty were aware of the institutional grievance/ complaints committee, interestingly, almost half of the trainees were not aware of its existence. It is essential to create awareness and constantly reinforce the existence of an approachable institutional grievance committee with no fear of repercussions. We suggest to do so by enquiring about the trainee's well-being through e mails once in a couple of months from the committee with information on redressal avenues and requesting them to revert back if there is a need to discuss any issue.

•  Distributing administrative responsibility equally

Trainees look up to their faculty and/or seniors and try to follow in their path. These role models must actively endorse gender equality and seek to set examples. We have a mentorship program within the department which is voluntary and requires the mentee to regularly interact and connect with their mentor. We have recently modified our mentorship program to involve both, a senior and junior faculty mentor for each trainee opting for a mentor, so as to help establish a more comfortable and approachable platform to the mentee to interact with their mentors.

•  Distributing administrative responsibility equally

Women faculty tend to handle interactions more compassionately (34) and are hence often tasked with the responsibility of allocating resident rotation duties and serving as “first responders” when trainees are distressed. It is likely that they could feel overburdened by this added responsibility, creating dissatisfaction and a sense of discrimination. A more gender equitable distribution of such responsibilities may be beneficial.

## Conclusion

Gender discrimination can present in subtle or overt fashion in surgical departments and requires active sustained efforts to allow both genders to feel equally empowered. It is necessary to ensure that the work environment remains conducive for each individual to perform to their optimum capability without deleterious effects on their mental well-being. Establishing a system to objectively evaluate gender equity while avoiding stereotyping for certain roles can help minimize GD.

## Data Availability

The original contributions presented in the study are included in the article/Supplementary Material, further inquiries can be directed to the corresponding author/s.
